# A Novel Molecularly Imprinted Sensor Based on CuO Nanoparticles with Peroxidase-like Activity for the Selective Determination of Astragaloside-IV

**DOI:** 10.3390/bios13110959

**Published:** 2023-10-28

**Authors:** Guo-Ying Chen, Ling-Xiao Chen, Jin Gao, Chengyu Chen, Jianli Guan, Zhiming Cao, Yuanjia Hu, Feng-Qing Yang

**Affiliations:** 1 Department of Pharmaceutical Engineering, School of Chemistry and Chemical Engineering, Chongqing University, Chongqing 401331, China; 20221801017@stu.cqu.edu.cn (G.-Y.C.); 20175534@cqu.edu.cn (L.-X.C.); 2 Jiaheng Pharmaceutical Technology Co., Ltd., Zhuhai 519000, China; noveltcm@163.com (J.G.); chenchengyu@fusenpharma.com (C.C.); fusenxinyao@fusenpharma.com (J.G.); alexcao@fusenpharma.com (Z.C.); 3 Henan Fusen Pharmaceutical Co., Ltd., Nanyang 473000, China; 4 State Key Laboratory of Quality Research in Chinese Medicine, Institute of Chinese Medical Sciences, University of Macau, Macao 999078, China

**Keywords:** molecularly imprinted polymer, astragaloside-IV, peroxidase-like activity, CuO nanoparticles, nanozyme

## Abstract

In this work, dopamine (DA) was polymerized on the surface of CuO nanoparticles (CuO NPs) to form a molecularly imprinted polymer (MIP@PDA/CuO NPs) for the colorimetric detection of astragaloside-IV (AS-IV). The synthesis process of MIP is simple and easy to operate, without adding other monomers or initiators. CuO NPs has high peroxidase (POD)-like activity that can catalyze the oxidation of 3,3′,5,5′-tetramethylbenzidine (TMB) to generate oxidized TMB (OxTMB) in the presence of H_2_O_2_, having a maximum ultraviolet-visible (UV-Vis) absorption peak at 652 nm. The AS-IV can specifically bind to the surface imprinted cavities and prevent the entry of TMB and H_2_O_2_, which will lead to the inhibition of the catalytic reaction. Therefore, a new approach based on the POD-like activity of MIP@PDA/CuO NPs for AS-IV detection was developed with a linear range from 0.000341 to 1.024 mg/mL. The LOD and LOQ are 0.000991 and 0.000341 mg/mL, respectively. The developed method can accurately determine AS-IV in Huangqi Granules and different batches of Ganweikang Tablets, which are similar to the results measured by HPLC-ELSD and meet the requirements of Chinese Pharmacopoeia (2020 edition) for the amount of AS-IV in Huangqi Granules. The combination of MIP with CuO NPs not only endows the detection of AS-IV with high selectivity and reliability, but also expands the application of nanozymes in the detection of small-molecule compounds that have weak UV absorption, and do not have reducibility or oxidation properties.

## 1. Introduction

Nanozymes, functional nanomaterials with enzyme-catalyzed properties, are simple to prepare, low-cost, highly stable, controllable in size, and adjustable in function [1], including metal compounds [2], organic compounds [3], and polymers [4]. With the recent and rapid development of nanomaterials, nanozymes can be classified into three main categories based on their applications in analytical chemistry: hydrolases [5], oxidoreductases [6], and transferases mimics [7]. Oxidoreductases mimics can be further classified into superoxide dismutase (SOD)-, oxidase (OXD)-, catalase (CAT)-, and peroxidase (POD)-like activities [8], which are widely used in environmental monitoring and biological analysis. Nanozymes that are applied for the optical detection of small-molecule compounds are mainly based on their POD- and OXD-like activities, which can act on H_2_O_2_ to generate free radicals or directly react with substrates to produce changes in absorbance or fluorescence intensity. Most of these small-molecule compounds are substrates of the nanozyme or substances with strong oxidizing or reducing activity. For example, Liu et al. [9] reported that the homogeneous copper nanoclusters exhibited excellent tetra enzyme-like activities, including POD-, CAT-, SOD-, and ascorbic acid oxidase-mimic activities, which can result in the detection of glutathione and ascorbic acid that have reducibility and can affect the generation of products. However, it is very challenging to achieve efficient and selective detection of compounds that are not substrates of nanozymes, have weak ultraviolet-visible (UV-Vis) absorption, and are free of reducibility and oxidation.

Molecularly imprinted polymers (MIPs) are synthesized through copolymerizing functional monomers and crosslinkers in the presence of the template molecule to form 3D polymeric networks. After removing the template from the networks, the specific recognition sites remain, which are capable of binding specifically to the templates in size, shape, and functional groups [10], and can realize the high selectivity detection of the template molecule. In addition, MIPs also have excellent stability and a long service life and can resist harsh environmental conditions, including changes in pH and temperature, in practical applications [11]. Some studies have focused on the combination of MIPs with different chemical technologies, such as colorimetry [12]. MIP can shield interference from other substances and extract template molecules from complex samples to improve detection selectivity. The colorimetric method based on enzyme-catalyzed reaction can amplify the detection signal and further improve sensitivity based on the absorbance change of products [13]. For example, Guo et al. [14] synthesized a novel molecularly imprinted sensor based on PtCu bimetallic nanoparticles deposited on poly (styrene sulfonate) functionalized graphene with POD-like activity for puerarin determination. The results indicate that the established method can achieve highly selective detection of puerarin, and can be used in the analysis of complex samples. With a structure similar to puerarin, including nobiletin and luteolin, the compounds showed no obvious influence on the catalytic reaction. Notably, CuO nanoparticles (CuO NPs) with POD-like activity are considerably stable and possess an almost unchanged catalytic activity [15]. In addition, dopamine (DA) and polydopamine (PDA), containing many active functional groups (such as amino and phenolic hydroxyl groups), can be deposited as versatile and strongly adhesive films on solid surfaces and react with substances containing thiols or amino groups [16]. Thus, this study intended to develop a colorimetry method based on the POD-like activity of CuO NPs, and the detection selectivity was realized through the MIP prepared using PDA coated on the surface of CuO NPs.

Astragaloside-IV (AS-IV) is one of the main active ingredients in *Astragalus membranaceus* (Huangqi in Chinese), which has many pharmacological effects such as regulating immunity, anti-inflammatory, protecting the heart, antitumor, and antifibrosis [17,18]. Due to its weak absorption capacity in the ultraviolet light region (200–400 nm), AS-IV cannot be directly detected by UV-Vis spectroscopy. Therefore, the amount of AS-IV in *A. membranaceous*, Astragalus formula granules, and other prescription preparations listed in the Chinese Pharmacopoeia (2020 edition), was determined through high-performance liquid chromatography-evaporative light scattering detection (HPLC-ELSD) [19]. Although HPLC-ELSD is widely used, it has some limitations, such as requiring expensive instruments, complicated sample pretreatment, and being time-consuming. The operation is relatively complex and unsuitable for rapid analysis. In this work, MIP@PDA/CuO NPs were synthesized through DA aggregated on the surface of CuO NPs, which have high POD-like activity and can catalyze the oxidation of 3,3′,5,5′-tetramethylbenzidine (TMB) to generate oxidized TMB (OxTMB) in the presence of H_2_O_2_. A selective colorimetric sensor was designed and successfully applied in the determination of AS-IV in Huangqi Granules and Ganweikang Tablets with satisfactory results, similar to that obtained through HPLC-ELSD analysis. The results meet the requirements of the Chinese Pharmacopoeia (2020 edition) for the amount of AS-IV in Huangqi Granules. It is worth mentioning that by using this strategy, it is possible to efficiently and selectively detect compounds that are not substrates of nanozymes, have weak UV-Vis absorption, and do not have reducibility or oxidation properties.

## 2. Materials and Methods

### 2.1. Materials and Reagents

Copper (II) acetate and acetic acid were obtained from Chongqing Chuandong Chemical Group Co., Ltd. (Chongqing, China). DA was purchased from Shanghai Aladdin Biochemical Technology Co., Ltd. (Shanghai, China). Sodium hydroxide (NaOH) and H_2_O_2_ were obtained from Chengdu Chron Chemicals Co., Ltd. (Chengdu, China). TMB was obtained from Shanghai Adamas Reagent Co., Ltd. (Shanghai, China). AS-IV (≥98.0%) was purchased from Macklin Biochemical Technology Co., Ltd. (Shanghai, China). Forsythia glycoside (≥98.0%) was purchased from Shanghai Bidepharm Co., Ltd. (Shanghai, China). Glycyrrhizin (98.0%) was purchased from Shanghai Yien Chemical Technology Co., Ltd. (Shanghai, China). Astragaloside I (AS-I) (≥98.0%), ursolic acid (≥98.5%), and oleanolic acid (≥99.0%) were purchased from Chengdu PUSH Bio-technology Co., Ltd. (Chengdu, China). Huangqi Granules (15 g/bag) were purchased from Chongqing Wanjiayan Pharmacy (Chongqing, China). Ganweikang Tablets (batch numbers 210202, 210201, 200102, 200801, and 201201) were purchased from Henan Fushen Pharmaceutical Co., Ltd. (Nanyang, China).

### 2.2. Instruments

The UV-Vis analysis was carried out on a UV-5500 PC spectrophotometer (Shanghai Metash Instruments Co., Ltd., Shanghai, China). The centrifugation of the samples was carried out through an L420 tabletop low-speed centrifuge (Hunan Xiang Yi Laboratory Instrument Development Co., Ltd., Changsha, China). The temperature-controlling process was achieved through an SHZ-82 shaker oscillator (Jintan Chengxi Zhengrong Experimental Instrument Factory, Changzhou, China). The structure of the materials was characterized by using a JSM-7600F field-emission scanning electron microscopy (SEM) (JEOL Co., Ltd., Tokyo, Japan). A certain amount of the materials was dispersed in ethanol, placed on a silicon wafer, dried, and sprayed gold to improve its conductivity before measurement. The images and element compositions of the materials were obtained through a Talos F200S transmission electron microscopy (TEM) (Thermo Fisher Scientific, Prague, Czech Republic). A certain amount of the materials was dispersed in aqueous solution, placed on a nickel mesh, dried, and measured. In addition, the ultrapure water used throughout this study was purified using the ATSelem 1820A water purification system (Antesheng Environmental Protection Equipment Co., Ltd., Chongqing, China).

### 2.3. Synthesis of CuO NPs

The preparation method for CuO NPs was described in a previous report [20]. First, 150 mL of copper acetate aqueous solution (0.2 M) and 0.5 mL of acetic acid were mixed into a conical flask and sonicated until they were completely dissolved. The solution was heated in a water bath until boiling, then 10 mL of NaOH (0.04 g/mL) was added, and a large amount of black precipitate was generated immediately. Finally, the solution was cooled to room temperature and centrifuged at 3800 rpm for 10 min. The supernatant was removed and the precipitate was washed with ethanol three times and dried in a 35 °C oven overnight.

### 2.4. Synthesis of MIP@PDA/CuO NPs

AS-IV (5 mg) and ethanol (4 mL) were mixed in a glass bottle and sonicated until they were completely dissolved. Then, 20 mg of CuO NPs was added to the above solution and sonicated for 2 min to disperse evenly. Subsequently, 25.8 mg of DA was added and stirred with magnetic force for 12 h. The reacted solution was centrifuged to remove the supernatant, and the precipitate was washed with ethanol twice and then dispersed in a methanol–water (9:1, *v*/*v*) solution and shaken in a 50 °C shaker for 1 h (150 rpm) to remove the template molecule AS-IV. The product was centrifuged and washed once with ethanol and water. Finally, the product was dispersed in 2, 1, and 0.5 mL of ultrapure water to obtain different material concentrations of A, B, and C, respectively, and then placed in a 4 °C refrigerator before use. NIP@PDA/CuO NPs were synthesized through the same process without adding the template molecule AS-IV.

### 2.5. Preparation of Huangqi Granules and Ganweikang Tablets Solution

Huangqi Granules (10 g) and ultrapure water (50 mL) were mixed into a beaker and sonicated for 30 min. Then, ultrapure water was added to compensate for the weight lost during the extraction. After shaking and filtering, 25 mL of filtrate was extracted four times with 25 mL of *n*-butanol. Then, the *n*-butanol extract solutions were merged, 30 mL of ammonia water was added, and the washing solution (upper layer solution) was discarded. This was repeated three times. Then, the *n*-butanol solution was separated and steamed for 25 min at 60 °C to remove the solvent. Finally, the residue was dissolved in 5 mL of ethanol solution and transferred to a 10 mL centrifuge tube to obtain the tested solution of the Huangqi Granules. The above steps were repeated to prepare the test solution of Ganweikang Tablets.

### 2.6. Peroxidase-like Catalytic Activity of MIP@PDA/CuO NPs and Detection of AS-IV

The POD-like activity of MIP@PDA/CuO NPs in the presence or absence of AS-IV solution was determined by the catalytic reaction of TMB with H_2_O_2_. Briefly, 50 μL of MIP@PDA/CuO NPs and 200 μL of ethanol or AS-IV were added into a 2 mL tube and incubated in a 40 °C shaker for 5 min (150 rpm); then, 10 μL of H_2_O_2_ (3 M) and 200 μL of TMB (20 mM) were added. After incubating for 10 s, the solution was centrifuged with a handheld mini centrifuge for 1 min, and the absorbance of the supernatant at 652 nm was recorded by a UV-Vis spectrometer. Each sample was measured three times. Under optimal conditions, the amount of AS-IV in the Huangqi Granules and different batches of Ganweikang Tablets was determined. For the recovery study, the prepared Huangqi Granule and Ganweikang Tablets solutions were diluted by 120 and 80 times, respectively. Then, different concentrations of AS-IV reference compound solution (the final concentrations of 0.000341, 0.512, and 1.024 mg/mL) were added to the diluted solutions, respectively. The spiked recoveries % = (found – original)/added × 100%, where found and original are the concentrations of AS-IV measured with and without the addition of AS-IV reference compound solution, and added is the concentration of added AS-IV.

### 2.7. HPLC-ELSD Detection of AS-IV

The AS-IV reference compound solutions with different concentrations were prepared for HPLC-ELSD analysis (Shimadzu, Japan, LC-20A). The amount of AS-IV in the Huangqi Granules and Ganweikang Tablets was determined through the calibration curve of the reference compound. By adding different concentrations of AS-IV reference compound solution (the final concentrations of 0.00156, 0.394, and 0.788 mg/mL) to the prepared Huangqi Granule and Ganweikang Tablets solutions, the spiked recoveries were measured and calculated as the previously mentioned formula. The AQ-C18 column (150 × 4.6 mm, 5 µm) was used for separation. Mobile phases A and B are ultrapure water and acetonitrile, respectively. The gradient elution procedure was as follows: 0–15 min, 10–100% B; 15–16 min, 5–100% B. Other conditions: the flow rate was 1.0 mL/min, the column temperature was 30 °C, the injection volume was 20 μL, and the ELSD instrument temperature was set at 50 °C.

## 3. Results and Discussion

### 3.1. Characterization of CuO NPs and MIP@PDA/CuO NPs Nanocomposites and Feasibility of the Established Method for the Detection of AS-IV

**Figure 1:**
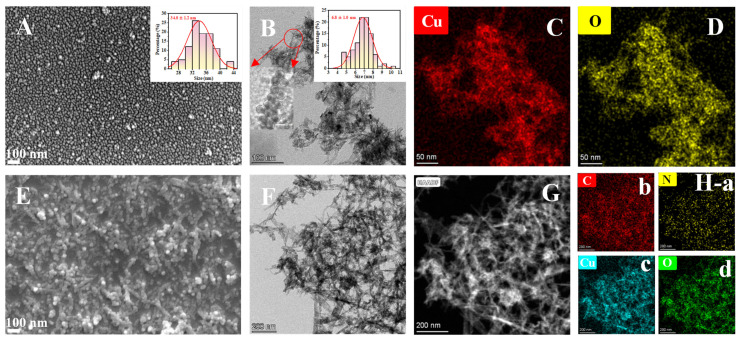
SEM images of CuO NPs (**A**) and MIP@PDA/CuO NPs (**E**); TEM images of CuO NPs (**B**) and MIP@PDA/CuO NPs (**F**,**G**); EDS results of CuO NPs (**C**,**D**) and MIP@PDA/CuO NPs (**H**-a,b,c,d). The inserted picture in A is the size distribution diagram of the CuO NPs. The inserted pictures in B are the size distribution diagram and partial enlarged image of the CuO NPs.

The morphology of the as-prepared nanocomposites was characterized using SEM and TEM. As shown in Figure 1A, CuO NPs possess a spherical morphology with an average particle size of about 34.0 ± 1.2 nm (the inserted picture). However, the TEM images provide a result (Figure 1B) with the mean size of 6.8 ± 1.0 nm (the inserted picture), which is basically consistent with that of the reference using the same synthesis method [20], and the CuO NPs contain Cu and O elements according to the EDS mapping profiles (Figure 1C,D). It is worth mentioning that the possible reason for the inconsistent particle size results obtained from SEM and TEM is the difference in the sample preparation process as to whether to spray gold or not [21,22]. Compared to the CuO NPs, MIP@PDA/CuO NPs are more clustered and unevenly dispersed (Figure 1E,F), which contain Cu, O, C, and N elements (Figure 1G,H). Elements C and N may mainly come from PDA and AS-IV. These results demonstrate that MIP@PDA/CuO NPs was successfully synthesized.

**Figure 2:**
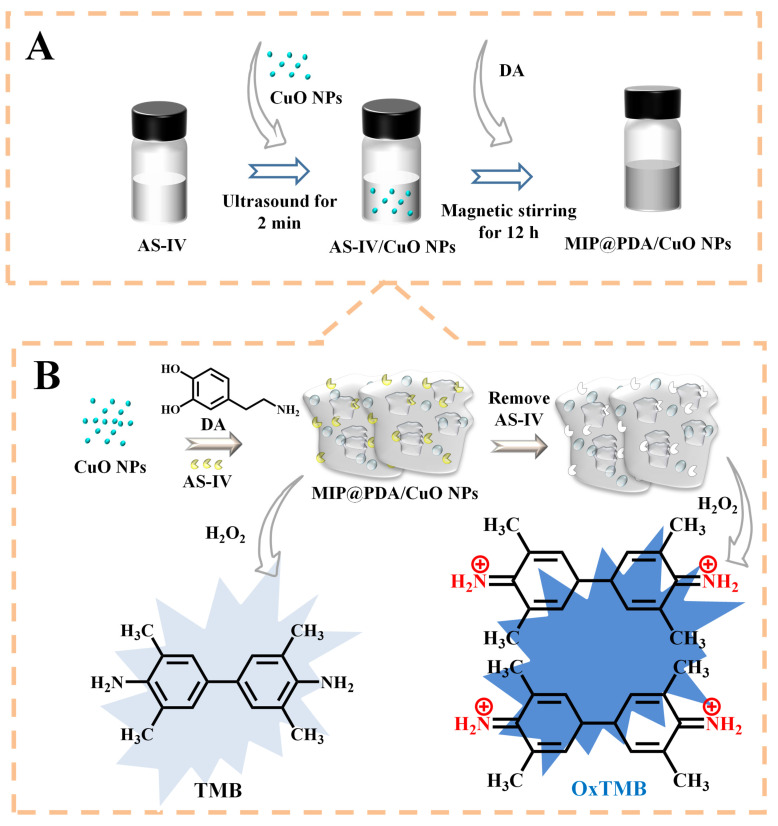
Schematic diagram of the preparation of MIP@PDA/CuO NPs nanocomposite (**A**) and its application in the detection of AS-IV (**B**). AS-IV: astragaloside-IV; DA: dopamine.

**Figure 3:**
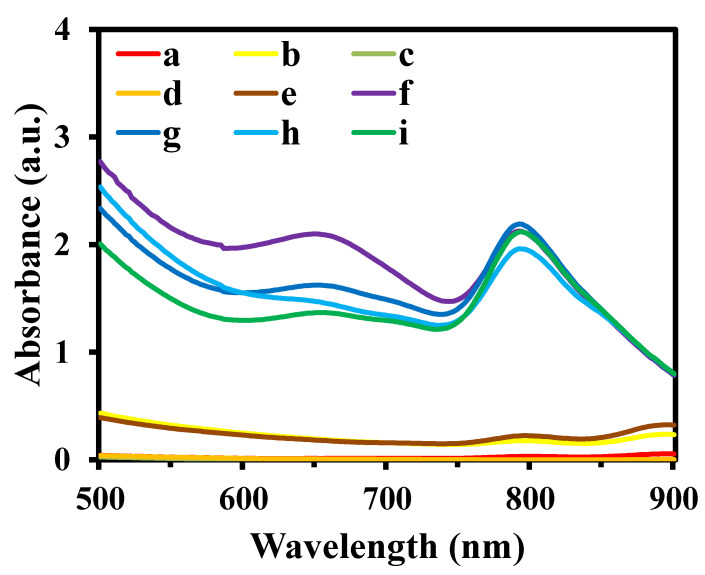
UV-Vis absorption spectra of different reaction systems. TMB + H_2_O_2_ (a), MIP@PDA/CuO NPs + TMB (b), AS-IV (c), TMB (d), NIP@PDA/CuO NPs + TMB (e), MIP@PDA/CuO NPs + H_2_O_2_ + TMB (f), NIP@PDA/CuO NPs + H_2_O_2_ + TMB (g), MIP@PDA/CuO NPs + AS-IV + H_2_O_2_ + TMB (h), and NIP@PDA/CuO NPs + AS-IV+ H_2_O_2_ + TMB (i).

The MIP@PDA/CuO NPs were synthesized through a simple magnetic stirring method formed by the interactions between CuO NPs and DA (Figure 2A). The CuO NPs, which can accelerate the conversion process of DA to PDA, serve as a solid matrix for DA to polymerize on their surface. Without AS-IV, H_2_O_2_ can pass through the imprinted cavities and contact the POD-like nanocomposite to generate radicals, resulting in the oxidation of TMB. In the presence of AS-IV, it can specifically bind to the surface imprinted cavities and prevent the entry of TMB and H_2_O_2_ through the cavities, which leads to the inhibition of the catalytic reaction of CuO NPs (Figure 2B). Therefore, a selective colorimetric sensor for AS-IV detection was established based on the absorbance changes of the product OxTMB. As shown in Figure 3, the solutions of TMB + H_2_O_2_ (a), MIP@PDA/CuO NPs + TMB (b), AS-IV (c), TMB (d), and NIP@PDA/CuO NPs + TMB (e) show no significant UV-Vis absorption peak at 652 nm. The MIP@PDA/CuO NPs + H_2_O_2_ + TMB (f) solution exhibits an obvious UV-Vis absorption peak, and the absorbance decreases with the addition of AS-IV (h). The absorbance changes of NIP@PDA/CuO NPs + H_2_O_2_ + TMB in the absence (g) and presence (i) of AS-IV are not obvious. These results indicate that the simulated enzyme activity of MIP@PDA/CuO NPs is POD- rather than OXD-like activity. Moreover, the successfully synthesized MIP@PDA/CuO NPs nanocomposites can be used for the colorimetric detection of AS-IV.

### 3.2. Study of Reaction Variables

**Figure 4:**
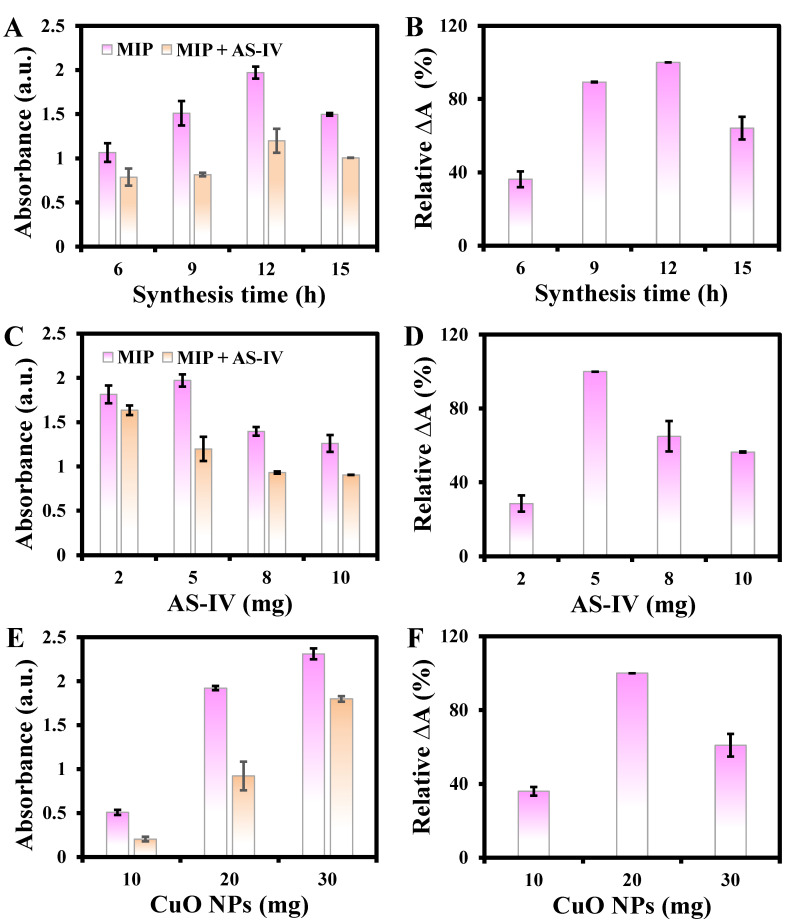
Effects of synthesis time (**A**,**B**), amounts of AS-IV (**C**,**D**) and CuO NPs (**E**,**F**) on the relative ΔA%.

Several factors, such as the synthesis time, amount of template AS-IV, CuO NPs, and DA, elution time, concentration of material, TMB, and H_2_O_2_, reaction temperature, and reaction time (1 and 2), may affect the performance of MIP@PDA/CuO NPs. Thus, these experimental conditions were systematically studied. Relative ΔA%, which is the percentage of absorbance decrease due to the presence of AS-IV over the maximum absorbance decrease, was used to evaluate the performance of MIP@PDA/CuO NPs (ΔA = A_0_ − A_1_, where A_0_ and A_1_ refer to the absorbance produced by the reaction in the absence and presence of AS-IV, respectively). The synthesis time affected the polymerization of DA and the synthesis of MIP. The absorbance increased as the synthesis time increased from 6 to 12 h (Figure 4A). The maximum ΔA% is obtained at the synthesis time of 12 h (Figure 4B), which was selected for the following applications. When the synthesis time was 15 h, the decreased absorbance was probably due to the fact that more AS-IV participated in the formation of MIP as the synthesis time increased. However, when the elution time was determined, the template molecules AS-IV eluted per unit time were limited, resulting in limited exposed cavities. The amount of template AS-IV is an important factor in the preparation of MIP. The absorbance increased and then decreased with the amount of AS-IV ranging from 2 to 10 mg (Figure 4C). The ΔA% was the highest when the amount of AS-IV was 5 mg (Figure 4D), which was selected for the following studies. The decrease in ΔA% is probably due to excessive AS-IV affecting the reaction between CuO NPs and DA, further affecting the synthesis of MIP. When the amount of CuO NPs ranged from 10 to 30 mg, the absorbance gradually increased (Figure 4E), and the ΔA% was highest at 20 mg (Figure 4F), which was chosen for subsequent experiments. A possible reason for the decrease in ΔA% at 30 mg is that excess CuO NPs may not participate in the formation of MIP with DA, allowing for its direct catalytic reactions.

**Figure 5:**
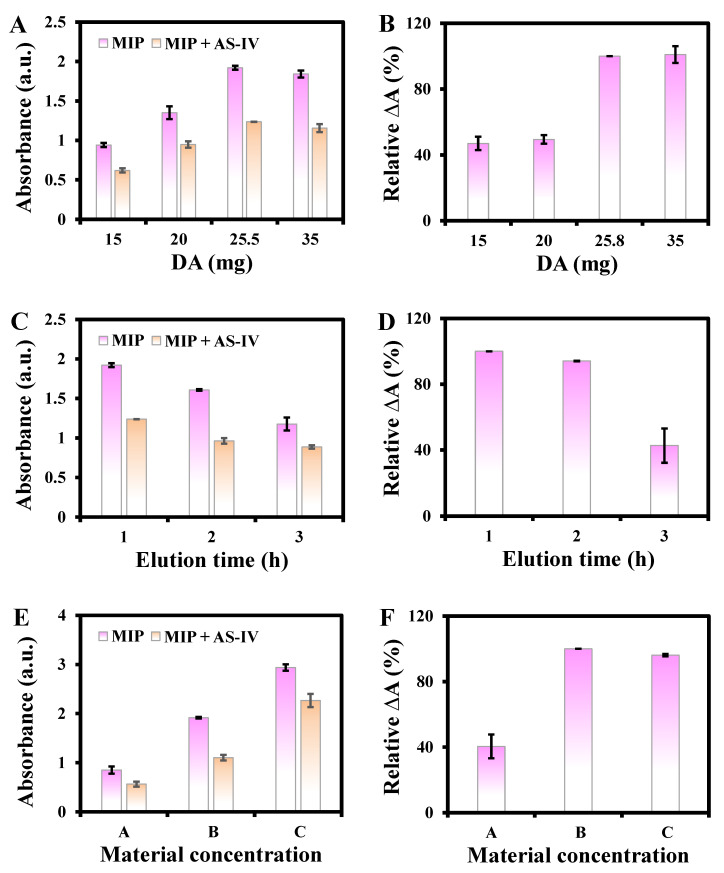
Effects of amount of DA (**A**,**B**), elution time (**C**,**D**), and material MIP@PDA/CuO NPs concentration (**E**,**F**) on the relative ΔA%. For the MIP@PDA/CuO NPs concentrations, please refer to Section 2.4.

When the amount of DA increased from 15 to 35 mg, the absorbance and ΔA% gradually increased and remained steady (Figure 5A,B). Thus, the amount of DA at 25.5 mg was selected for further reactions. The effect of elution time was also optimized. The absorbance decreased with increasing elution time from 1 to 3 h (Figure 5C), and the highest ΔA% was obtained at 1 h (Figure 5D), which was chosen for further experiments. In addition, material concentration is also a significant factor. The results show that when the material (MIP@PDA/CuO NPs) concentration is too high or low, the absorbance and ΔA% decrease (Figure 5E,F); thus, the material concentration of B was chosen for the following studies. The possible reason is that the excessive MIP@PDA/CuO NPs tend to form thicker MIP membranes, which make it difficult for the covered template molecules to be eluted, strongly preventing H_2_O_2_ from entering the cavity. However, a low concentration of MIP@PDA/CuO NPs provides insufficient molecular imprinting cavities, resulting in fewer specific recognition sites.

**Figure 6:**
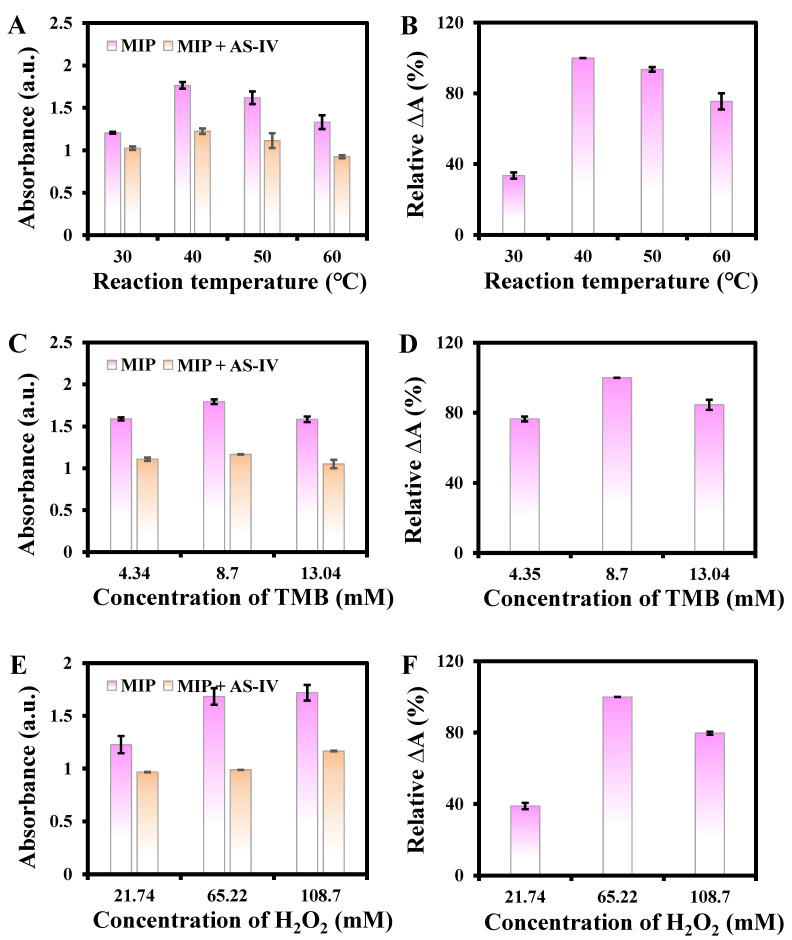
Effects of reaction temperature (**A**,**B**), concentrations of TMB (**C**,**D**) and H_2_O_2_ (**E**,**F**) on the relative ΔA%.

When the reaction temperature was changed from 30 to 60 °C, the maximum ΔA% was obtained at 40 °C (Figure 6A,B), which was selected for further study. Moreover, the effects of TMB and H_2_O_2_ concentrations were also investigated. The absorbance changed with an increase in TMB concentrations from 4.34 to 13.04 mM (Figure 6C). The maximum ΔA% was obtained at 8.7 mM (Figure 6D), which was chosen for subsequent studies. The system displays a decrease in ΔA% at a TMB concentration of 13.04 mM, and a possible reason may be the excessive TMB affecting the reaction between H_2_O_2_ and the material. The absorbance gradually increased and remained steady with the increase of H_2_O_2_ concentrations from 21.74 to 108.7 mM (Figure 6E). The maximum ΔA% was obtained at 65.22 mM (Figure 6F), which was selected for subsequent experiments.

**Figure 7:**
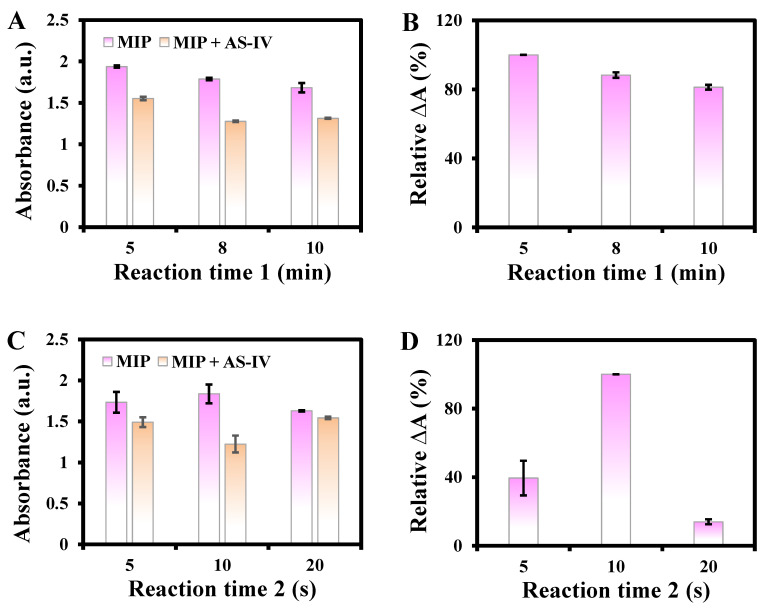
Effects of reaction time 1 (**A**,**B**) and 2 (**C**,**D**) on the relative ΔA%.

Reaction time 1 and 2 refer to reactions of MIP@PDA/CuO NPs + AS-IV, and MIP@PDA/CuO NPs + AS-IV + H_2_O_2_ + TMB, respectively. As reaction time 1 varied from 2 to 10 min, the absorbance in the absence of AS-IV showed no obvious changes, and that in the presence of AS-IV gradually decreased (Figure 7A). The maximum ΔA% was obtained at 5 min (Figure 7B). Thus, reaction time 1 of 5 min was selected for further studies. In addition, the ΔA% in reaction time 2 was highest at 10 s (Figure 7C,D), which was chosen for the following studies. Therefore, the studied reaction conditions for the detection of AS-IV based on MIP@PDA/CuO NPs were as follows. The synthesis time was 12 h, the amounts of AS-IV, CuO NPs, and DA were 5 mg, 20 mg, and 25.5 mg, respectively, elution time was 1 h, material concentration was B, reaction temperature was 40 °C, concentrations of TMB and H_2_O_2_ were 8.7 and 65.22 mM, respectively, and reaction time 1 and 2 were 5 min and 10 s, respectively.

### 3.3. Detection of AS-IV Based on MIP@PDA/CuO NPs

**Figure 8:**
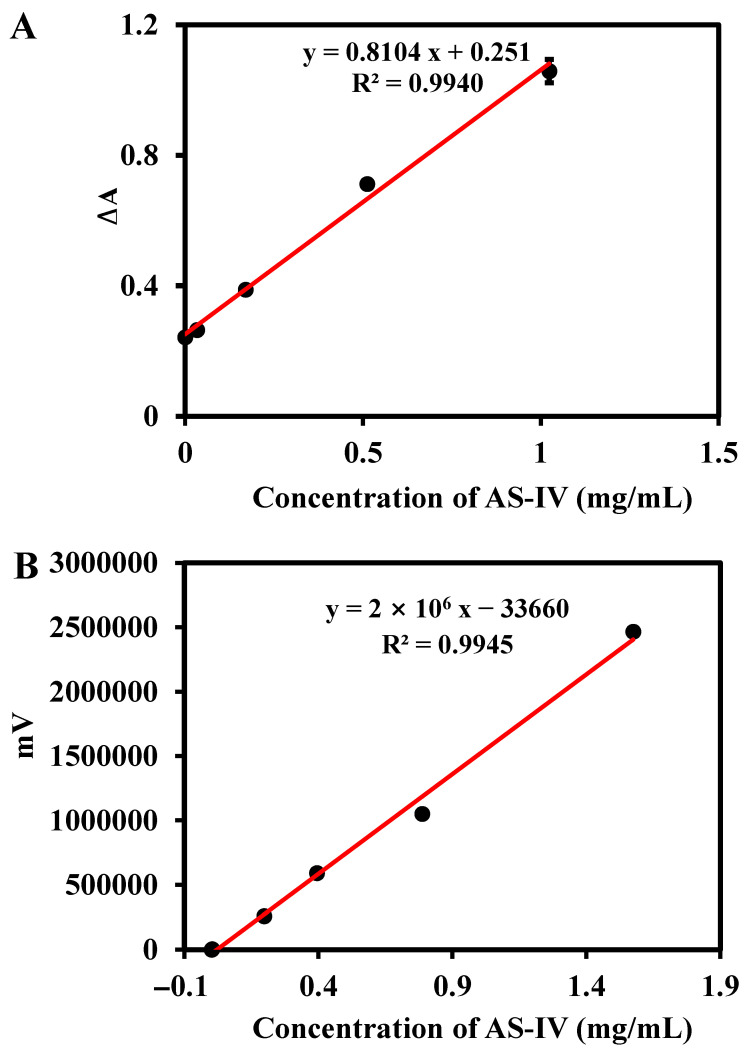
The linear relationship curves of AS-IV determined through UV-Vis (**A**) and HPLC-ELSD (**B**) analysis.

The gradual addition of AS-IV resulted in a continuous decrease in absorbance at 652 nm, indicating that it occupied more cavities to prevent interaction between H_2_O_2_ and CuO NPs. As shown in Figure 8A, the change of ΔA% (652 nm) is linearly proportional to the AS-IV concentrations in the range from 0.000341 to 1.024 mg/mL with the limit of detection (LOD) and limit of quantitation (LOQ) values of 0.000991 and 0.000341 mg/mL, respectively. The obtained linear equation was y = 0.8104x + 0.2510 (R^2^ = 0.9940). In addition, HPLC-ELSD was used to determine AS-IV, and a linear relationship between its concentration and the response signal was established. As shown in Figure 8B, with the concentrations of AS-IV varying from 0.00156 to 1.576 mg/mL, a good linear relationship was observed. The linear equation was y = 2 × 10^6^ x − 33660 (R^2^ = 0.9945). The LOD (S/N = 3) and LOQ (S/N = 10) were 0.0083, and 0.17 mg/mL, respectively. There are few reports on the detection of AS-IV, especially based on inhibiting enzyme catalytic activity. Compared with existing methods for AS-IV detection, such as the high-performance thin-layer chromatographic (linear range: 1.01–10.10 mg/mL) [23] and ultra-high-performance liquid chromatography (linear range: 0.0000784–0.00392 mg/mL, LOD: 0.00157 × 10^−3^ mg/mL, LOQ: 0.00784 × 10^−3^ mg/mL) [24], this proposed method has a comparable linear range for AS-IV determination. It is worth noting that although the sensitivity of the developed sensor using UV-Vis is limited, the established sensor provides a simple method to detect AS-IV without the need of an expensive instrument, which has a certain potential in the routine analysis of practical samples.

**Figure 9:**
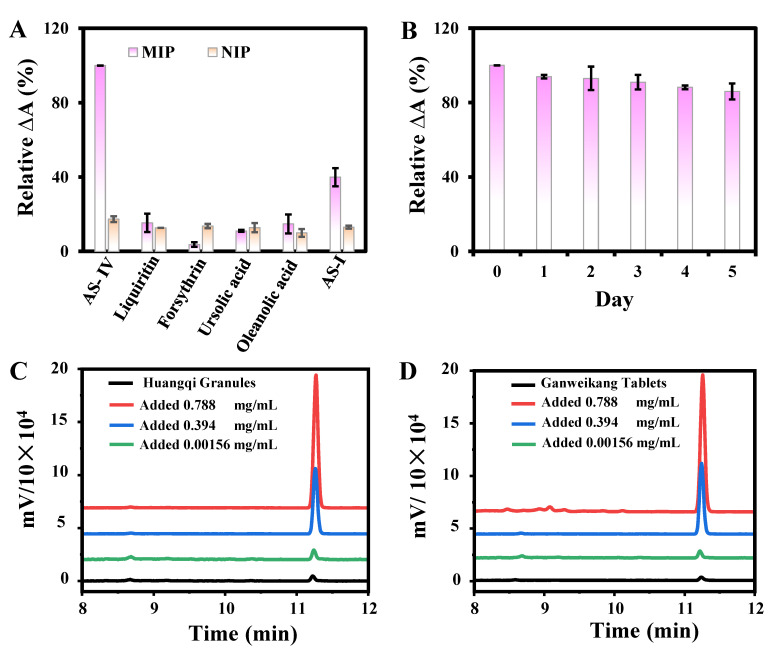
The selectivity of MIP@PDA/CuO NPs toward AS-IV and other structural analogues (**A**). Storage stability of MIP@PDA/CuO NPs (**B**). HPLC-ELSD chromatograms of the actual samples of Huangqi Granules (**C**) and Ganweikang Tablets (**D**).

Some substances with similar structures or functional groups to AS-IV that may exist in the crude extracts of Ganweikang Tablets and Huangqi Granules were selected for the selectivity experiments, including liquiritin, forsythrin, ursolic acid, oleanolic acid, and AS-I. The concentrations of AS-IV and other substances were 0.171 and 0.341 mg/mL, respectively. As shown in Figure 9A, the effects of liquiritin, forsythrin, ursolic acid, and oleanolic acid on the reaction were negligible. Huangqi Granules and Ganweikang Tablets may simultaneously contain AS-I, -II, -III, and -IV, whose difference lies in the number of COOHs in the structure. For the treatment of actual samples, according to the method for the quantitative determination of AS-IV in Huangqi Granules in the Chinese Pharmacopoeia (2020 edition), adding large amounts of ammonia can convert AS-I, II, and III into AS-IV. Therefore, the influence of AS-I can also be ignored. The results clearly indicate the high selectivity of the proposed method for AS-IV detection. In addition, the storage stability of the composite material MIP@PDA/CuO NPs was investigated. As shown in Figure 9B, the same batch of materials was stored in a refrigerator at 4 °C for 5 days, and its activity was 85% of its initial activity, indicating that it has excellent storage stability.

### 3.4. AS-IV Detection in Huangqi Granules and Ganweikang Tablets

To verify the feasibility of this method in complex sample analysis, the standard addition method was used to determine AS-IV in Huangqi Granules and Ganweikang Tablets using UV-Vis and HPLC-ELSD. As shown in Table 1, the UV-Vis and HPLC-ELSD spiked recoveries were between 80.0% and 113.1%, and the RSD was less than 5.9%. The amount of AS-IV in the Huangqi Granules was measured to be 0.34 mg/g by UV-Vis, which is close to the HPLC-ELSD analysis results (0.31 mg/g) and meet the requirements of the Chinese Pharmacopoeia (2020 edition) for the amount of AS-IV in Huangqi Granules (≥0.3 mg/g). In addition, as shown in Figure 9C,D, the HPLC-ELSD chromatograms of the spiked crude extracts of the Huangqi Granules and Ganweikang Tablets show a peak at 11.23 min. As the spiked sample concentrations ranged from 0.00156 to 0.788 mg/mL, the peaks gradually increased. Finally, these two methods were used to determine the amount of AS-IV in different batches of Ganweikang Tablets. As shown in Table 2, the amount of AS-IV detected in different batches is similar, which indicates that the developed colorimetric method can be used for the detection of AS-IV in complex samples with high reliability and accuracy.

## 4. Conclusions

In this work, an analytical method for the selective detection of AS-IV was established by enwrapping CuO NPs with MIP and combining their high POD-like activity. In the MIP synthesis process, no other monomers or initiators were added. Furthermore, CuO NPs can accelerate the conversion process of DA to PDA, and they can serve as a solid matrix for DA to polymerize on its surface. This method displays high selectivity and reliability for the detection of AS-IV, which is neither a substrate nor a substance with strong oxidizing or reducing activity, by using the POD-like catalytic activity of nanozyme directly. Although the colorimetric method has limited sensitivity, the operation is simple and the cost is lower compared to other methods such as HPLC-ELSD, which provides an effective detection method for compounds with poor UV absorption. Subsequent research can focus on the improvement of detection sensitivity by adding other monomers and initiators to synthesize more completed MIP and preparing composite nanomaterials with multienzyme activity by introducing other elements and materials for doping or post-modification.

## Figures and Tables

**Table 1:** Spiked recovery of AS-IV in Huangqi Granules and Ganweikang Tablets.

UV-Vis					HPLC-ELSD			
Sample	Added (mg/mL)	Found (mg/mL)	Recovery (%)	RSD (*n* = 3, %)	Added (mg/mL)	Found (mg/mL)	Recovery (%)	RSD (*n* = 3, %)
Huangqi Granules	0	0.000555 ^a^	-	1.0	0	0.0605	-	1.2
	0.000341	0.000856	90.9	2.9	0.00156	0.0620	91.5	4.7
	0.512	0.494	93.8	3.0	0.394	0.374	80.9	5.9
	1.024	0.856	83.5	5.8	0.788	0.714	80.0	4.3
Ganweikang Tablets	0	0.000352 ^b^	-	0.9	0	0.0244	-	1.5
	0.000341	0.000745	113.1	2.2	0.00156	0.0261	109.0	1.3
	0.512	0.584	108.9	4.9	0.394	0.342	83.8	3.6
	1.024	1.059	102.6	0.8	0.788	0.715	89.0	5.0

^a,b^: The prepared Huangqi Granule and Ganweikang Tablets solutions were diluted by 120 and 80 times, respectively, to fit the linear range of the UV-Vis method.

**Table 2:** Amount of AS-IV in different batch numbers of Ganweikang Tablets.

Ganweikang Tablets	UV-Vis (μg/g)	HPLC-ELSD (μg/g)
210202	122.93 ± 1.4	120.84 ± 0.8
210201	123.40 ± 1.0	121.20 ± 0.7
200102	122.15 ± 1.1	121.62 ± 1.2
200801	123.11 ± 0.9	120.26 ± 1.1
201201	122.68 ± 1.6	120.51 ± 0.7

## Data Availability

Not applicable.
